# T Cell Protein Tyrosine Phosphatase in Glucose Metabolism

**DOI:** 10.3389/fcell.2021.682947

**Published:** 2021-06-29

**Authors:** Ya-nan Wang, Shiyue Liu, Tingting Jia, Yao Feng, Xin Xu, Dongjiao Zhang

**Affiliations:** ^1^Department of Implantology, School and Hospital of Stomatology, Cheeloo College of Medicine, Shandong University, Jinan, China; ^2^Shandong Key Laboratory of Oral Tissue Regeneration, Jinan, China; ^3^Shandong Engineering Laboratory for Dental Materials and Oral Tissue Regeneration, Jinan, China; ^4^Department of Periodontology, School and Hospital of Stomatology, Cheeloo College of Medicine, Shandong University, Jinan, China

**Keywords:** T cell protein tyrosine phosphatase, protein tyrosine phosphatase non-receptor 2, glucose metabolism, insulin signaling pathway, leptin signaling pathway

## Abstract

T cell protein tyrosine phosphatase (TCPTP), a vital regulator in glucose metabolism, inflammatory responses, and tumor processes, is increasingly considered a promising target for disease treatments and illness control. This review discusses the structure, substrates and main biological functions of TCPTP, as well as its regulatory effect in glucose metabolism, as an attempt to be referenced for formulating treatment strategies of metabolic disorders. Given the complicated regulation functions in different tissues and organs of TCPTP, the development of drugs inhibiting TCPTP with a higher specificity and a better biocompatibility is recognized as a promising therapeutic strategy for diabetes or obesity. Besides, treatments targeting TCPTP in a specific tissue or organ are suggested to be considerably promising.

## Introduction

Protein tyrosine kinases and phosphatases critically impact numerous biological activities (Denu et al., 1996; Humphrey et al., 2015). TCPTP, also known as protein tyrosine phosphatase non-receptor 2 (PTPN2) refers to a classical non-receptor protein tyrosine phosphatase, which was initially cloned from T-cell cDNA library (Cool et al., 1989; Mosinger et al., 1992; Andersen et al., 2001). TCPTP has two variants, i.e., TC45 and TC48. To be specific, TC45 is located in nuclear, and TC48 is located in endoplasmic reticulum (Bussieres-Marmen et al., 2014). TC45 is expressed in both human and mice, whereas TC48 is expressed only in human. In response to cytokine stimulation, TC45 shuttles from the nucleus to cytoplasm (Tiganis et al., 1999). TCPTP is capable of regulating pathways in glucose metabolism (Gurzov et al., 2014; Sharp et al., 2015; Wiede et al., 2019), inflammation control (Spalinger et al., 2018, 2020; Hsieh et al., 2020; Parlato et al., 2020), cancer progression (Stuible et al., 2008; Manguso et al., 2017; LaFleur et al., 2019; Wiede et al., 2020) and other biological processes by dephosphorylating distinct substrates (Bourdeau et al., 2005), including Janus activated kinase (JAK), signal transducer and activator of transcription (STAT), receptor tyrosine kinases (RTKs) and others. In the present review, the structure, main substrates (JAK/STAT and RTKs), biological functions of TCPTP, as well as its regulatory role in glucose metabolism are summarized, as an attempt to be referenced for developing treatment strategies of metabolic disorders.

## Structure and Expression of TCPTP

**FIGURE 1:**
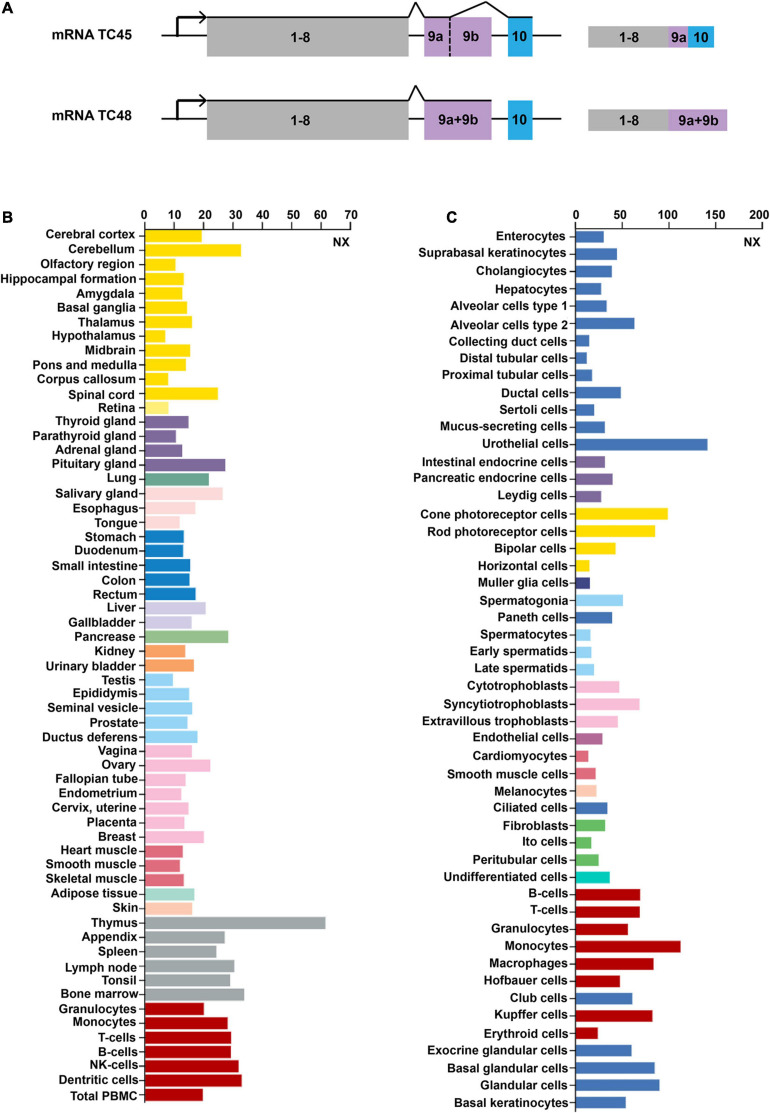
Gene structures and expression profiles of TCPTP. **(A)** Gene structures of TCPTP (modified according to reference Bussieres-Marmen et al., 2014). Exon 9a is identified in both TC45 and TC48, while exon 9b only presents in TC48. TC45 is transcribed by exon 9a and exon 10, while TC48 is transcribed by exon 9a + 9b, but without exon 10. **(B)** Expression profiles of TCPTP in different normal tissues. **(C)** Expression profiles of TCPTP in various normal cells. Data are available on https://www.proteinatlas.org/. NX represents the consensus normalized expression level.

Sakaguchi et al. (1992) initially detected *Tcptp* located on 18p11.2-p11.3 in humans. As impacted by the alternative splicing at the 3′ end of the gene, two isoforms of TCPTP are generated. TC45 composes 387 amino acids translated from ten exons. TC48 consists of 415 amino acids translated from nine exons. According to Figure 1A, exon 9a is identified in TC45 and TC48, while exon 9b only presents in TC48. TC45 is transcribed by exon 9a and exon 10, while TC48 is transcribed by exon 9a + 9b, but without exon 10 (Lorenzen et al., 1995). The exon 9a existing in both isoforms harbors the Nuclear localization signal (NLS) sequence, which can assure the protein located to the nucleus. However, a predominant hydrophobic sequence exists in the 9b exon of TC48. Such a hydrophobic sequence is an NLS inhibitor, capable of preventing TC48 from being located to the nuclear (Lorenzen et al., 1995).

TCPTP is widely distributed in different tissues or cells and we have provided an overview of the mRNA expression files of TCPTP. As shown in Figure 1B, TCPTP is highly expressed in the thymus, pancreas and cerebellum tissues. As shown in Figure 1C, TCPTP is highly expressed in the urothelial cells, monocytes, and cone photoreceptor cells.

## Main Substrates of TCPTP

**FIGURE 2:**
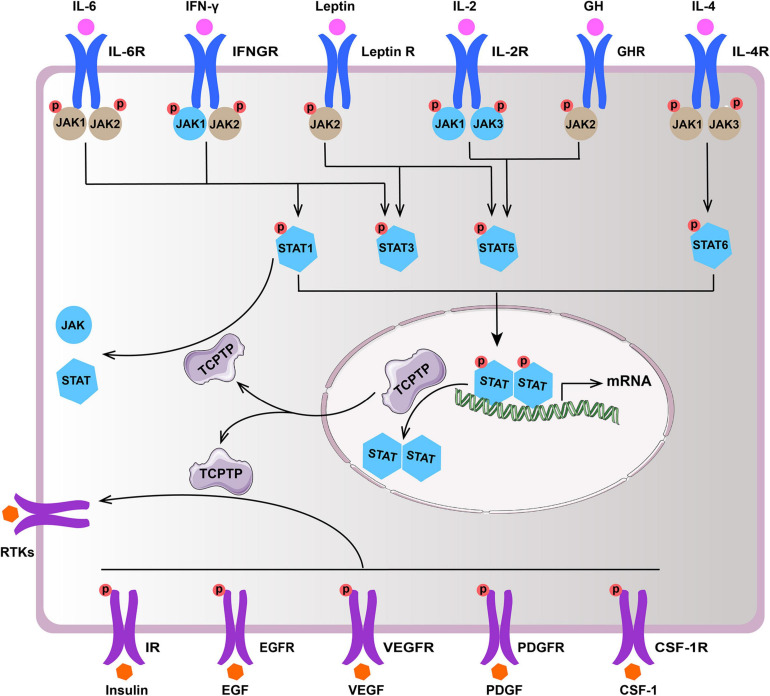
Main substrates of TCPTP (modified according to reference Bourdeau et al., 2005). Upon ligand binding, the receptor of IL-2, IL-4, IL-6, IFN-γ, GH, and leptin associated JAKs and STATs become activated. TCPTP can dephosphorylate JAK1 and JAK3, as well as STAT1, STAT3, STAT5, and STAT6 (molecules in blue), whereas it is not identical in different cytokine signaling according to summary of existing literatures. Besides, TCPTP is capable of regulating diverse signaling pathways by dephosphorylating various RTKs (e.g., IR, EGFR, VEGFR, PDGFR, and CSF-1R). TCPTP, T cell protein tyrosine phosphatase; IL-6, interleukin-6; IL-6R, interleukin-6 receptor; IFN-γ, interferon-γ; IFNGR, interferon-γ receptor; Leptin R, leptin receptor; IL-2, interleukin-2; IL-2R, interleukin-2 receptor; GH, growth hormone; GHR, growth hormone receptor; IL-4, interleukin-4; IL-4R, interleukin-4 receptor; JAK, Janus activated kinase; STAT, signal transducer and activator of transcription; RTKs, receptor tyrosine kinases; IR, insulin receptor; EGF, epidermal growth factor; EGFR, epidermal growth factor receptor; VEGF, vascular endothelial growth factor; VEGFR, vascular endothelial growth factor receptor; PDGF, platelet-derived growth factor; PDGFR, platelet-derived growth factor receptor; CSF-1, colony-stimulating factor-1; CSF-1R, colony-stimulating factor-1 receptor.

TCPTP regulates diverse signaling pathways by dephosphorylating distinct substrates [e.g., Janus activated kinase (JAK), Signal Transducer and Activator of Transcription (STAT), and Receptor Tyrosine Kinases (RTKs)]. This review summarizes the main substrates (JAK/STAT and RTKs) of TCPTP (Figure 2 and Table 1).

**TABLE 1:** Main substrates and dephosphorylating sites of TCPTP.

**Substrates**	**Dephosphorylating sites**	**Related signaling**	**References**
JAK1	Y1022 and Y1023	IFN-γ, IL-2 signaling	Simoncic et al., 2002
JAK3	Not reported	IL-2 signaling	Simoncic et al., 2002
STAT1	Y701	IL-6, IL-7, IFN-γ signaling	ten Hoeve et al., 2002
STAT3	Y705	IL-6, IFN-γ, leptin signaling	Yamamoto et al., 2002; Zhang Y. et al., 2018
STAT5	Y694 and Y699	IL-2, GH, leptin signaling	Yu et al., 2000
STAT6	Not reported	IL-4 signaling	Lu et al., 2007; Lu et al., 2005
IR	Y1162 and Y1163	Insulin signaling	Galic et al., 2003
EGFR	Y992 and Y1068	EGF/EGFR/PI3K signaling	Scharl et al., 2010b
VEGFR	Y1054/1059,Y1214 and Y996	VEGF/VEGFR signaling	Mattila et al., 2008
PDGFR	Y1021 and Y751	PDGF/PDGFR signaling	Kramer et al., 2020
CSF-1R	Y807	CSF-1/CSF-1R signaling	Zhang et al., 2019

### JAK/STAT

#### JAK1 and JAK3

JAK1 and JAK3 are specific substrates of TCPTP (Kleppe et al., 2010; Luo et al., 2018; Zhang P. et al., 2018). The dephosphorylating sites refer to Y1022 and Y1023 in JAK1, while the dephosphorylating site of JAK3 has not been reported. Bone marrow-derived macrophages from *Tcptp*^–/–^ mice showed hyper-phosphorylation of JAK1 after the interferon-γ (IFN-γ) treatment (Simoncic et al., 2002). Moreover, TCPTP interacts with JAK1 and JAK3 after activation of interleukin-2 (IL-2) receptor (Simoncic et al., 2002).

#### STAT1, STAT3, STAT5, and STAT6

First, TCPTP accounts for STAT1 dephosphorylating (ten Hoeve et al., 2002), and it is involved in downregulation of interleukin-6 (IL-6) signaling (Twohig et al., 2019), interleukin-7 (IL-7) signaling (Pike et al., 2017), and IFN-γ signaling (Heinonen et al., 2009). Second, it has been evidenced that TCPTP is capable of dephosphorylating STAT3 at Y705 site (Zhang Y. et al., 2018) and then attenuate IL-6 signaling (Yamamoto et al., 2002), IFN-γ signaling (Scharl et al., 2010a), and leptin signaling (Loh et al., 2011). Third, TCPTP dephosphorylates STAT5 (Y694 in STAT5a and Y699 in STAT5b) and subsequently attenuates IL-2 signaling, growth hormone signaling and leptin signaling that are associated with T cell differentiation, energy regulation, etc. (Yu et al., 2000; Aoki and Matsuda, 2002). Lastly, STAT6 is also a reported substrate for TCPTP. It is evidenced that TCPTP knockdown will increase IL-4-induced STAT6 signaling in B-cell lymphomas (Lu et al., 2005, 2007).

### Receptor Tyrosine Kinases

#### Insulin Receptor (IR)

IR refers to a transmembrane protein tyrosine kinase. IR can phosphorylate its downstream substrates [e.g., the Insulin Receptor Substrate-1 (IRS-1)] upon binding insulin (Belfiore et al., 2017). TCPTP can downregulate insulin signaling by dephosphorylating IR (Galic et al., 2003; Tiganis, 2013).

#### Epidermal Growth Factor Receptor (EGFR)

EGFR can be activated by EGF family directly, which has been identified as a specific substrates of TCPTP (Tiganis et al., 1998). As revealed from in-depth studies, the knockdown of TCPTP can facilitate EGFR tyrosine phosphorylation. The residues dephosphorylated by TCPTP are identified as Y992 and Y1068 in Hela cells and human colonic T84 epithelial cells (Mattila et al., 2005; Scharl et al., 2010b).

#### Vascular Endothelial Growth Factor Receptor (VEGFR)

TCPTP significantly limits the VEGF/VEGFR signaling and consequently preventing excessive angiogenesis. TCPTP dephosphorylates VEGFR in a phosphosite-specific manner and inhibits its kinase activity (Mattila et al., 2008). Baek et al. (2018) reported that epidermal-specific knockout of TCPTP can improve UVB-induced epidermal cell survival by dephosphorylating VEGFR and then regulating VEGFR/JNK signaling.

#### Platelet-Derived Growth Factor Receptor (PDGFR)

TCPTP can control the embryo differentiation and fibroblasts proliferation by regulating the PDGF/PDGFR signaling pathway (Persson et al., 2004; Karlsson et al., 2006). Kramer et al. reported that TCPTP knockdown could increase PDGFR phosphorylation at Y751 and Y1021, thereby leading to an enhanced downstream signaling and increased growth rates of fibroblasts (Kramer et al., 2020).

#### Colony-Stimulating Factor-1 Receptor (CSF-1R)

Colony-Stimulating Factor 1 (CSF-1) regulates the survival and differentiation of mononuclear phagocytes by binding with CSF-1R (Stanley and Chitu, 2014). According to Simoncic et al. (2006) TCPTP could negatively regulate the CSF-1/CSF-1R signaling. Zhang et al. (2019) reported that TCPTP could significantly inhibit alveolar bone resorption by dephosphorylating CSF-1R at Y807 site.

## Main Functions of TCPTP

**FIGURE 3:**
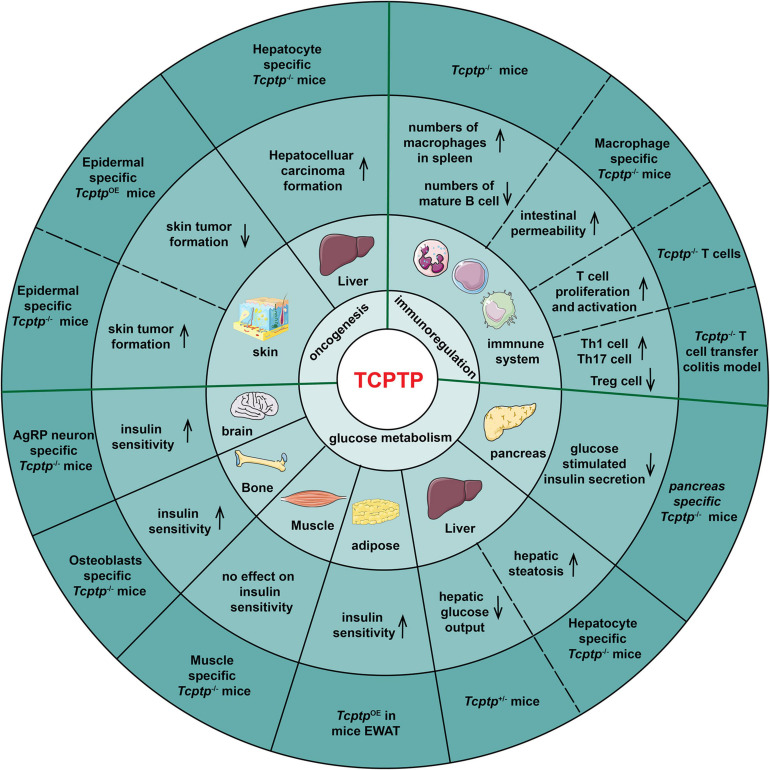
TCPTP-mediated biological processes in different tissues and organs. The experimental models, the resulting effects, the tissues or organs and the biological functions of TCPTP are presented from the outside to the inside of the pie chart. OE indicates “overexpression.”

According to Figure 3, the manifestations in glucose metabolism, immunoregulation and oncogenesis in *Tcptp* knockout models are summarized.

### TCPTP in Glucose Metabolism

The effect of TCPTP on glucose metabolism varies with tissues. In pancreas, pancreas-specific *Tcptp*^–/–^ mice exhibits impaired glucose tolerance and Glucose-Stimulated Insulin Secretion (GSIS) when challenged with high fat feeding (Xi et al., 2015). In liver, TCPTP ablation results in enhanced growth hormone signaling, weight gain, insulin resistance and hepatic steatosis sequentially (Gurzov et al., 2015). The fasted blood glucose and hepatic glucose output decrease in *Tcptp*^+/–^ mice (Fukushima et al., 2010). In adipose, TCPTP overexpression in Epididymal White Adipose Tissue (EWAT) reverses the high Th17/Treg and M1/M2 macrophage ratios significantly, improving insulin resistance of diabetic mice (Li et al., 2018). In muscle, muscle-specific *Tcptp* deficiency does not impact insulin signaling and glucose homeostasis (Loh et al., 2012). In bone tissue, mice lacking TCPTP in osteoblasts showed an increased bone resorption, osteocalcin bioactivity, and insulin sensitivity (Zee et al., 2012). In brain, TCPTP deletion in Agouti-Related Peptide (AgRP) neurons facilitates insulin sensitivity (Dodd et al., 2018).

### TCPTP in Immunoregulation

Overall, TCPTP can inhibit inflammation. *Tcptp*^–/–^ mice die soon after birth as impacted by increased numbers of macrophages in spleen and severe systemic inflammatory disease (You-Ten et al., 1997; Heinonen et al., 2004; Scharl et al., 2010a). Spalinger et al. (2020) demonstrated that macrophage-specific TCPTP deficiency increased intestinal permeability via IL-6 release. Besides, *Tcptp*^–/–^ mice developed B cell deficiency, which was because pre-B cells failed to transit to immature B cells (You-Ten et al., 1997; Doody et al., 2009). For T cells, TCPTP negatively regulates T cell proliferation and activation (Rhee and Veillette, 2012). Hyper-phosphorylation of the activated Lck and reduction of TCR threshold were proved to account for increased T cell activation in *Tcptp*^–/–^ T cells (Wiede et al., 2011). The infusion of *Tcptp*^–/–^ CD4^+^ T cells to colitis mice led to an almost threefold increase of Th1 cell frequency, a twofold increase of Th17 cell frequency and in contrast, a threefold decrease of Tregs frequency (Spalinger et al., 2015).

### TCPTP in Oncogenesis

As demonstrated from the generation of epidermal-specific TCPTP-deficient mice, TCPTP suppresses skin tumor formation by down-regulating STAT3 and AKT signaling (Morales et al., 2019). Furthermore, the numbers of skin tumors decrease significantly in epidermal-specific TCPTP-overexpression mice (Kim et al., 2020). TCPTP deletion in hepatocytes promotes non-alcoholic steatohepatitis as well as hepatocellular carcinoma in obese mice (Grohmann et al., 2018). Besides, TCPTP can inhibit oncogenesis of breast cancer (Shields et al., 2013; Veenstra et al., 2019), most of the hematopoietic malignancies (Kleppe et al., 2011; Pike and Tremblay, 2016) and glioblastoma (Navis et al., 2010).

## The Related Signaling Pathways of TCPTP in Glucose Metabolism

### Insulin Signaling Pathway

**FIGURE 4:**
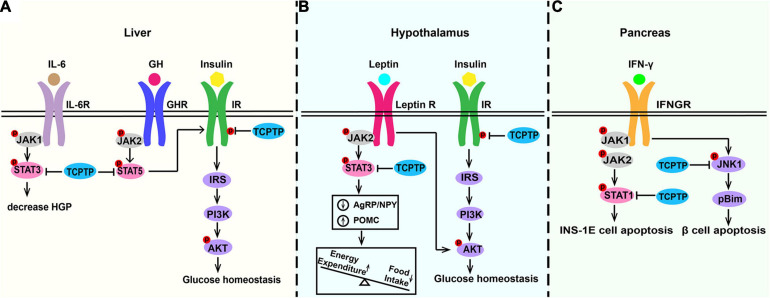
Molecular pathways of TCPTP in regulating glucose metabolism in **(A)** liver, **(B)** hypothalamus, and **(C)** pancreas. In liver, TCPTP can dephosphorylate IR and STAT3 to inhibit insulin signaling and IL-6 signaling separately to promote hepatic glucose output. In hypothalamus, TCPTP may act as the principal phosphatase to attenuate insulin and leptin signaling by inhibiting insulin-induced AKT phosphorylation and leptin-induced STAT3 signaling. In pancreas, TCPTP modulates IFN-induced β cell death by dephosphorylating pro-apoptotic protein Bim via JNK1. Furthermore, TCPTP is capable of effectively suppressing the IFN-γ/STAT1 signaling pathway in INS-1E cells. TCPTP, T cell protein tyrosine phosphatase; IL-6, interleukin-6; IL-6R, interleukin-6 receptor; HGP, hepatic glucose production; GH, growth hormone; GHR, growth hormone receptor; IR, insulin receptor; Leptin R, leptin receptor; IFN-γ, interferon-γ; IFNGR, interferon-γ receptor; JAK, Janus activated kinase; STAT, signal transducer and activator of transcription; IRS, insulin receptor substrate; PI3K, phosphatidylinositol 3-kinase; AKT, protein kinase B; JNK1, c-Jun N-terminal kinase 1.

Insulin resistance characterized by insulin signaling pathway defects refers to the key pathological property exhibited by type 2 diabetes (Ono, 2019; Diane et al., 2021; He et al., 2021). Liver, brain, and muscle are the main insulin-sensitive organs (Pomytkin et al., 2018; Tokarz et al., 2018). In liver, insulin-induced phosphatidylinositol 3-kinase (PI3K)/Akt signaling pathway inhibits the expression of key enzymes of gluconeogenesis (Glucose-6-phosphatase and phosphoenolpyruvate carboxykinase) to decrease hepatic glucose production (Saltiel and Kahn, 2001). In TCPTP heterozygous deficiency mice, gluconeogenesis and hepatic glucose output were suppressed, the expression of gluconeogenic genes (glucose-6-phosphatase-alpha and phosphoenolpyruvate carboxykinase 1) were decreased, and the expression of lipogenic genes (sterol regulatory element-binding protein 1c and fatty acid synthase) were increased (Fukushima et al., 2010). According to Figure 4A, IR phosphorylation and PI3K/Akt signaling are up-regulated in hepatocytes derived from *Tcptp*^+/–^ mice (Fukushima et al., 2010). As shown in Figure 4B, TCPTP is the principal protein tyrosine phosphatase inhibiting insulin signaling in hypothalamus, especially in appetite-suppressing Proopiomelanocortin (POMC) neurons. TCPTP deficiency in POMC neurons can enhance insulin-induced AKT phosphorylation, improve glucose homeostasis, and prevent diet-induced obesity by increasing white adipose tissue browning and energy expenditure (Dodd et al., 2015). TCPTP deficiency in AgRP neurons improves systemic insulin sensitivity by regulating IR signaling, represses hepatic glucose production, and increases Brown Adipose Tissue (BAT) glucose uptake (Dodd et al., 2018). Though TCPTP regulates IR signaling in the liver and hypothalamus neurons, this does not extend to muscle. Muscle-specific lack of TCPTP do not exhibit any insulin sensitivity alterations in gastrocnemius muscle *in vivo* or in myoblasts *in vitro* (Loh et al., 2012). Besides, muscle glucose uptake and whole-body glucose tolerance are not impacted by TCPTP deficiency (Loh et al., 2012). What’s more, whole-body glucose homeostasis is also related to osteocalcin activity, which is regulated by insulin signaling in osteoblasts. Osteoblast-specific deletion of TCPTP promotes insulin sensitivity in an osteocalcin-dependent manner (Zee et al., 2012).

### Leptin Signaling Pathway

Leptin coordinates feeding, thermogenesis, as well as glucose homeostasis primarily via hypothalamic circuits (Adlanmerini et al., 2021; Montserrat-de la Paz et al., 2021; Perakakis et al., 2021; Pereira et al., 2021). The leptin pathway promotes anorexigenic neuropeptide POMC expression and represses the orexigenic Agouti-Related Peptide (AgRP)/Neuropeptide Y (NPY) expression (Zhang et al., 2015; Santoro et al., 2017). Leptin resistance in hypothalamic neurons plays a key role in exacerbating diet-induced obesity. As shown in Figure 4B, TCPTP is the key protein tyrosine phosphatase inhibiting leptin signaling in hypothalamus. As reported by existing studies, when the expression of hypothalamic TCPTP is up-regulated, the leptin signaling pathway will be attenuated, and the energy expenditure will decrease, thereby causing an increased weight gain (Loh et al., 2011). Hypothalamus-specific *Tcptp*^–/–^ mice can develop an enhanced leptin sensitivity, decreased food intake, reduced adiposity, and improved glucose metabolism (Dodd et al., 2019). TCPTP deletion in neuronal cells could not only promote the leptin-induced STAT3 signaling but also alter POMC and AgRP expression (Loh et al., 2011). However, TCPTP deletion in POMC neurons alone showed no effects on leptin signaling (Dodd et al., 2015).

### IFN-γ Signaling Pathway

Type 1 diabetes mellitus (T1DM) is attributed to pancreatic β cells destruction and insulin production insufficient (Katsarou et al., 2017; Li et al., 2017; Wei et al., 2019; Eizirik et al., 2020; Pang et al., 2020). According to Ounissi-Benkalha and Polychronakos (2008), Sharp et al. (2015), and Wiede et al. (2019), TCPTP is an important locus associated with T1DM. As revealed from in-depth studies, TCPTP can essentially ameliorate β cells from apoptosis caused by virus, dsDNA and IFN-γ (Op de Beeck and Eizirik, 2016). As shown in Figure 4C, TCPTP modulates IFN-induced β cell death by dephosphorylating pro-apoptotic protein Bim via JNK1 (Santin et al., 2011; Marroqui et al., 2014). In addition, siRNA targeting TCPTP exacerbates IFN-γ-induced apoptosis of INS-1E cells while double knockdown of TCPTP and STAT1 protects INS-1E cells against cytokine-induced apoptosis, which indicates that TCPTP can effectively suppress the IFN-γ/STAT1 signaling pathway in pancreatic cells to inhibit inflammation (Moore et al., 2009).

### GH/GHR/STAT5 Pathway

In liver, TCPTP ablation can facilitate growth hormone signaling and weight gain (Gurzov et al., 2015). TCPTP deletion in neuronal cells is capable of elevating the GH-induced STAT5 phosphorylation, decreasing the circulating GH levels, and increasing adiposity (Loh et al., 2011). Given the crosstalk between GH and insulin signaling (Huang et al., 2020), the systemic regulation effect of TCPTP is extremely complex, which requires in-depth investigations.

### IL-6/STAT3 Pathway

IL-6-instigated JAK/STAT3 signaling pathway has emerged as a significant mechanism for decreasing hepatic glucose production by inhibiting the expression of gluconeogenic genes (Inoue et al., 2004). Fukushima et al. (2010) reported that IL-6-induced STAT3 phosphorylation but not JAK1 was enhanced in *Tcptp*^+/–^ hepatocytes and hepatic glucose output was decreased in *Tcptp*^+/–^ mice. The results confirm the negative regulatory capacity of TCPTP acting directly on STAT3 in the liver to regulate gluconeogenesis.

## Therapeutic Strategies and Future Perspectives

IR and leptin signaling downregulation by TCPTP offers the possibility to develop TCPTP inhibitors as potential treatment strategy for T2DM or obesity. Sodium metavanadate refers to the first phosphatase inhibitors to be used clinically to improve human diabetes, which was initially used in 1899 (Heneberg, 2009). However, for the lack of specificity, they could cause unexpected side effects. In 2009, an extremely selective TCPTP inhibitor was synthesized, which showed 200-fold selectivity for TCPTP over other protein tyrosine phosphatases (Zhang et al., 2009). It is noteworthy that Loh et al. confirmed that administration of this inhibitor into cerebral ventricles could enhance leptin-induced STAT3 phosphorylation and energy expenditure in wild type mice (Loh et al., 2011) and up-regulate insulin-induced POMC expression by 2.5-fold (Dodd et al., 2015). Besides, some natural agents (e.g., celastrol) could promote weight loss in diet-induced obesity by inhibiting TCPTP in the hypothalamus (Kyriakou et al., 2018). Furthermore, Dodd et al. (2019) reported that the daily intranasal administration of the glucocorticoid antagonist RU486 down-regulating TCPTP expression could significantly help promote weight loss and improve glucose metabolism in obese mice.

Though the difficulties of TCPTP selectivity have been addressed, major challenges remain in tissues specific to the development of TCPTP inhibitors. This is of particularly importance for the role of TCPTP in hematopoietic development, immunoregulation and oncogenesis. The *in situ* delivery and sustained release of drugs targeting TCPTP in specific organs or tissues may be a promising development direction.

## Conclusion

On the whole, given the role of TCPTP in glucose metabolism by regulating insulin, leptin and other signaling pathways, this review considers that the development of specific and effective TCPTP inhibitors for individual cells, tissues or organs is a promising therapeutic strategy for diabetes or obesity. Moreover, the *in situ* delivery and sustained release systems of TCPTP inhibitors in specific tissues should be investigated in subsequent studies.

## Author Contributions

YW and SL drafted the initial manuscript. TJ and YF made substantial contributions to the acquisition of data. XX and DZ critically reviewed it for important intellectual content. All authors gave the final approval of the version to be published.

## Conflict of Interest

The authors declare that the research was conducted in the absence of any commercial or financial relationships that could be construed as a potential conflict of interest.
